# Supplementary material for: The Construction and Exploration of a Comprehensive MicroRNA Centered Regulatory Network in Foxtail Millet (Setaria italica L.)
Source: Front Plant Sci. 2022 May 6;13:848474. doi: 10.3389/fpls.2022.848474 (PMC9121102; doi:10.3389/fpls.2022.848474)
Supplement: Supplementary file 1 [file Data_Sheet_1.zip › Sit-miRN3027_Seita.5G026400.1_756_TPlot.pdf]

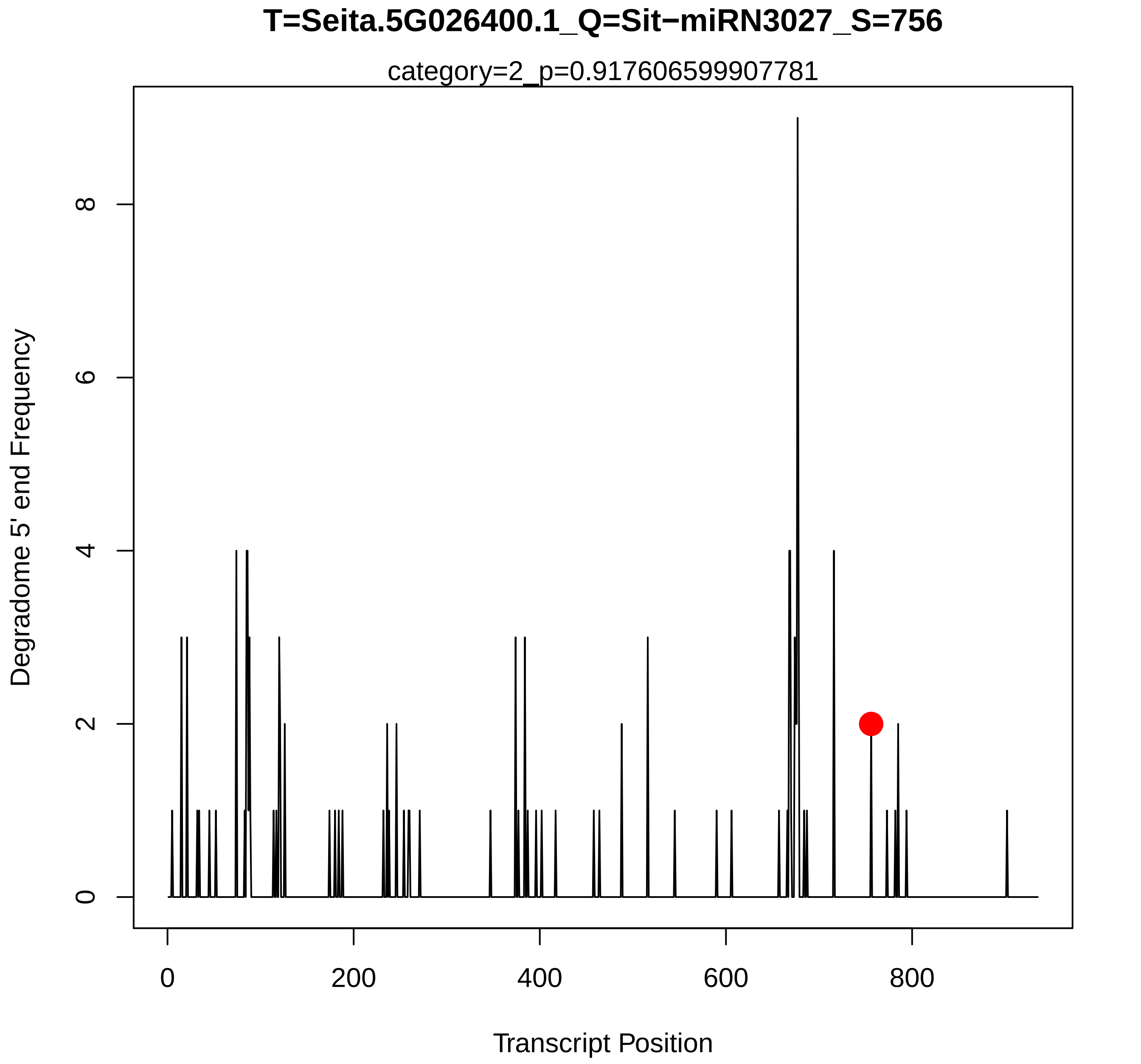

T=Seita.5G026400.1_Q=Sit−miRN3027_S=756
category=2_p=0.917606599907781
Degradome 5' end Frequency
0
2
4
6
8
0
200
400
600
800
Transcript Position
